# Radioprotective Activity and Synthesis of Siladithioacetals and Germadithioacetals Derived from *N*-substituted Naphthylethylimidazoline

**DOI:** 10.1155/MBD.2001.199

**Published:** 2001

**Authors:** Benoît Célariès, Ghassoub Rima, Louis Court, Claude Lion, Jean-Denis Laval

**Affiliations:** 1 Laboratoire d'Hétérochimie Fondamentale et Appliquée UMR 5069 du CNRS Université Paul Sabatier 118, route de Narbonne Toulouse cedex 4 F-31062 France; 2 Service de Radioprotection de I'EDF 22-28, rue Joubert Paris F-75009 France; 3 I.T.O.D.Y.S. Université de Paris VII Associé au CNRS 1, rue Guy de la Brosse Paris F-75005 France; 4 Unité de Radioprotection Centre de Recherche du Service de Santé des Armées 24, avenue des Maquis du Grésivaudan La Tronche cedex F-38702 France

## Abstract

A number of organosilicon and organogermanium derivatives and some related compounds including the *N*-substituted. 2-[1-naphthylmethyl]-2-imidazoline and 2-[1-(1-naphthyl)ethyl]-2-imidazoline have been prepared and the toxicity of some compounds have been determined in mice. In this paper we report the synthesis and the evaluation of the pharmacological activity of new organosilicon and organogermanium, compounds such as sila- and germadithioacetals derived from *N*-substituted naphthylimidazolne.


					Metal Based Drugs Vol. 8, No. 4, 2001
RADIOPROTECTIVE ACTIVITY AND SYNTHESIS OF SILADITHIOACETALS
AND GERMADITHIOACETALS DERIVED FROM N-SUBSTITUTED
NAPHTHYLETHYLIMIDAZOLINE
Benot C61ari6s,Ghassoub Rima*, Louis CoHrt2, Claude Lion
and Jean-Denis Laval4
1Laboratoire d'H6t6rochimie Fondamentale et Appliqu6e, UMR 5069 du CNRS, Universit6 Paul Sabatier,
118, route de Narbonne, F-31062 Toulouse cedex 4, France. Fax + (33) 5 61 55 82 04
< rima@chimie.ups-tlse.fr>
2Service de Radioprotection de I'EDF, 22-28, rue Joubert, F-75009 Paris, France
31.T.O.D.Y.S. Universit6 de Paris VII, Associ6 au CNRS, 1, rue Guy de la Brosse, F-75005 Paris, France
4Unit6 de Radioprotection, Centre de Recherche du Service de Sant6 des Arm6es, 24, avenue des Maquis du
Gr6sivaudan, F-38702 La Tronche cedex, France
ABSTRACT
A number of organosilicon and organogermanium derivatives and some related compounds
including the N-substituted 2-[1-naphthylmethyl]-2-imidazoline and 2-[1-(1-naphthyl)ethyl]-2-imidazoline
have been prepared and the toxicity of some compounds have been determined in mice. In this paper we
report the synthesis and the evaluation of the pharmacological activity of new organosilicon and
organogermanium compounds such as sila- and germadithioacetals derived from N-substituted
naphthylim dazo ne.
INTRODUCTION
Recently, it has been reported that medetomidine [1] is a new imidazole drug that possesses
selective and potent 2-adrenergic properties. 2-Adrenergic stimulation is known to mediate a variety of
biological actions including hypertension, sedation, antianxiety, analgesia, hypothermia, decreased salivary
secretions and mydriasis [2].
The selection of the naphthalene ring system was due to its presence in the potent (z-adrenergic
stimulant naphazoline. The presence of a methyl group attached at the benzylic position of medetomidine is
important for optimal (Zz-adrenergic activity [3].
Our group has already reported a considerable work in the field of the chemical radioprotection and
pharmacological activity of organosilicon and organogermanium compounds [4-16]. Several organometallic
classes derived from N-substituted naphthylmethylimidazoline showed a potent radioprotective activity.
Concerning the mechanism of the radioprotective activity of these compounds, it is well established
that the main mechanisms of radioprotection are tissual oxygen tension decrease, free radical scavenging and
hydrogen transfer [17]. However, the radioprotective action mechanism of naphthylmethylimidazoline
remains unclear. Some recent and preliminary investigations seem to indicate that at least in vitro, it has no
effect on lipidic peroxidation. Perhaps it acts through its vasoconstrictive effects, which can lower the tissual
oxygen supply and hence decrease the radiation-induced lesions.
Another possible explanation of excellent activity of naphthylmethylimidazoline, the first non-
sulfur radioprotective compound, is its evolution in vivo of the compound that forms anti-inflammatory
products of naphthyl-l-acetic acid type [18]. Further studies are necessary to determine the precise
mechanism of action.
Accordingly our objective, we have investigated a new series of organosilicon and
organogermanium compounds derived from N-substituted 2-[1-(1-naphthyl)ethyl]-2-imidazoline.
Experimental section
General procedures
All manipulations were performed under an inert atmosphere of argon using standard Schlenck,
glove box and high-vacuum-line techniques. All solvents used were freshly dried using standard techniques
and all glassware was oven-dried. IH-NMR spectra were recorded on a Bruker AC 80 spectrometer operating
,a 80.13 MHz (chemical shifts are reported in parts per million relative to internal Me4Si as reference) and
'"C-NMR spectra on an AC 200 spectrometer (50.32 MHz). The multiplicity of the 13C-NMR signals was
determined by the APT technique. Mass spectra under electron impact (EI) or chemical ionisation (CI/CH4)
conditions at 70 and 30 eV were obtained on Hewlett-Packard 5989 and Nermag RI0-10H spectrometers. IR
and UV spectra were recorded on Perkin-Elmer 1600 FT-IR and Lambda-17 spectrophotometers.
199
Ghassoub Rima et al. Radioprotective Activity ad Synthesis ofSiladithioacetals and Germadithio
Acetals Derivedfrom N-Substituted Napthylethylemidazoline
Table 1
Cpd
Physicochemical dataand analyses Of derivatives 1'12
Physical properties and elemental analyses
Yield
87
89
88
91
86
R='R n-C6Hi3; 3R CH3; M Ge
H-NMR (CDCI3): 0.88 (t, 6H, J 5.6 Hz, CH_3CH2); 1.09 1.56 (m, 26H,
(CH_2)5 and CH__3-CHS); 1.70 (d, 6H, J 6.8 Hz, CH__-CH); 2.38 2.76 (m, 2H,
CH3-CHS); 3.05 3.80 (m, 12H, CH__zN); 4.47 (q, 2H, J 6.8 Hz, CH-CH3);
7.12- 7.50 (m, 8H, C0H7); 7.63 7.95 (m, 4H, Cl0H7); 8.09 -8.35 (m, 2H,
CoHT). 3C-NMR (CDi3): 14.12" (CH3CH2); 19.07 (CH3-CH); 20.98 (CH3-
CH); 21.09 (CH-CH3); 22.58 (CH2Ge); 23.41 (CH3CH2); 24.69 (CH3CH2CH2);
31.10 (CH3CH2CH2CH2); 31.49 (CH2CH2Ge); 34.79 (CH-CH3); 50.28 (CH2N);
50.49 (CH2N); 52.69 (CH2N); 123.09 (aromatic CH); 124.72 (atom. CH);
125.61 (atom. CH); 126.02 (atom. CH); 126.18 (atom. CH); 127.87 (atom.
CH); 129.21 (atom. CH); 131.12 (atom. Cquat); 134.09 (arom. Cq,at); 137.10
(atom. Cqoa0; 167.21 (N-C=N). Analysis (C4sH6sN4S2Ge): Calcd. %: C, 68.85;
H, 8.!3; N, 6.69. Found %' C, 69.02; H, 8.17; N, 6.61.
=2R n-C6HI3; 3R H; M Ge
H-NMR (CDCI3): 0.86 (t, 6H, J 5.6 Hz, CH__3CH:); 1.02 1.47 (m, 20H,
(CH_.2)5); 1.68 (d, 6H, J 6.9 Hz, CH_.-CH); 2.36 2.84 (m, 4H, C_I_I_IS); 3.02
3.84 (m, 12H, CH_,zN); 4.43 (q, 2H, J 6.9 H C,[z[-CH3); 7.21 7.62 (m, 8H,
CoH7); 7.68- 7.93 (m, 4H, CoH7); 8.08 -.19 (m, 2H, Co_H7). 3C-NMR
(CDCI3): 14.22 (,H3CH2); 19.13 (CH3-CH); 21.26 (,G.HS); 22.70 (CH2Ge);
23.39 (CH3CH2); 24.65 (CH3CH2CH2); 31.21 (CH3CH/CH2CH2); 31.58
(CH2CH2Ge); 34.89 (CH-CH3); 50.34 (C_.H2N); 50.61 (CH2N); 52.74 (CH2N);
123.17 (aromatic CH); 124.86 (atom. CH); 125.74 (atom..G.H); 126.12 (atom.
CH); 126.23 (atom. C_H); 127.93 (atom. CH); 129.31 (atom. C_H); 131.23
(atom. Cqoat); 134.14 (atom. Cquat); 137.18 (atom. Cquat); 167.36 (N-C_=N).
Mass Spectrum: m/z 525 [M- 283]+. Analysis (C46H64N4S2Ge): Calcd. %: C,
6RS.2_@RH_,7.91; N, .93. Found %: C, 68.33; H, 8.00; N, 7.05.
i-CH; R CH3; M Ge
H-NMR (CDCI3): 0.88 (d, 12H, J 5.6 Hz, (CH_)2CH); 1.05 1.49 (m, 16H,
CH__2CH_.zCH and CH_.CHS); 1.69 (d, 6H, J 6.8 Hz, CH.-CH); 2.30 2.80 (m,
2H, CHS); 3.06 3.88 (m, 12H, CH_2N); 4.44 (q, 2H, J 6.7 Hz, CH-CH3);
7.22- 7.60 (m, 8H, CoH7); 7.65 7.92 (m, 4H, CoHT); 8.00 8.20 (m, 2H,
C,0H7). 3C-NMR (CDCI): 18.28 (,QH/Ge); 19.10 (CHS); 20.95 ((CH3)2CH);
21.08 (CH3-CH); 22.09 (CH3-CH); 30.52 ((CH3)z_C_CH); 32.25 (CH2CH); 34.83
(..H-CH3); 50.26 C.QH2N); 50.51 (CH2N); 52.65 C.QH2N); 124.01 (aromatic
CH); 125.41 (arom. CH); 125.73 (arom. CH); 126.49 (arom. CH); 126.64
(atom..G_H); 127.69 (atom. CH); 128.66 (arom. CH); 132.12 (atom. Cq,);
132.56 (atom. Cqu,,); 133.93 (arom. Cqu,,); 166.31 (N-C=N). Analysis
(C46H64N4S2Ge): Calcd. %: C, 68.26; H, 7.91; N, 6.93. Found %" C, 68.35; H,
7.94; N, 6.88.
3R
R ZR i-CsH; H; M Ge
H-NMR (CDCI3)" 0.87 (d, 12H, J 5.6 Hz, (CH.O.3)zCH); 1.06 1.44 (m, 10H,
CH__zCH__zCH); 1.71 (d, 6H, J 6.7 Hz, C]z[.3-CH); 2.36 2.77 (m, 4H, C_.I.S);
3.07 3.79 (m, 12H, C.H_zN); 4.45 (q, 2H, J 6.7 Hz, CH-CH3); 7.20 7.58
(m, 8H, C,oFIT); 7.64- 7.90 (m, 4H, C,0H7); 8.05 8.23 (m, 2H, C,o_.HT). 13C-
NMR (CDCI3)' 17.19 (CHzGe); 18.28 (CHzS); 19.10 ((CH3)zCH); 22.09 (CH3-
CH); 30.52 ((CH3)zCH); 32.25 (,C_.HzCH); 34.83 (.G.H-CH3); 50.26 (H,N);
50.51 (CHzN); 52.65 (CHzN); 124.01 (aromatic CH); 125.41 (arom. CH);
125.73 (atom. CH); 126.49 (atom. CH); 126.64 (atom. CH); 127.69 (arom.
CH); 128.66 (atom. CH); 132.12 (arom. Cqu,t); 132.56 (arom. Cq,t); 133.93
(atom. Cquat); 166.31 (N-C=N). Analysis (C44H6oN4SzGe): Calcd. %: C, 67.64;
H, 7.69; N, 7.17. Found %" C, 67.82; H, 7.74; N, 7.09.
'R p-CH3-C6H4; R CH3; R CH3; M Ge
H-NMR (CDCI3): 0.95 (s, 3H, CH_.3Ge); 1.35 (d, 6H, J 6.7 Hz, CH__3-CHS);
1.74 (d, 6H, J 6.8 Hz, CH__3-CH); 2.20-2.63 (m, 5H, p-CH_.3 and CHS); 3.08-
3.80 (m, 12 H, C.H_..N.); 4.51 (q, 2H, J 6.8 Hz, C_.H-CH3); 7.09-8.18 (rn, 18
H, C6H_4 and C,oH7). 'C-NMR (CDCI3): 9.02 (CH3-Ge); 20.11 (p-CH3); 20.75
(CH3-CH); 21.21 (CH3-CH); 21.65 (CH-CH3); 34.22 (CH-CH3); 45.92 (CHzN);
46.40 (CH.,N); 46.73 (.G.HzN); 123.20 (aromatic CH); 125.74 (atom. CH);
125.96 (atom. CH); 128.41 (atom. CH); 128.84 (atom. C_.H); 129.39 (atom.
CH); 129.47 (arom. CH); 129.70 (atom. C_.H); 130.02 (atom. CH); 131.79
200
Metal Based Drugs Vol. 8, No. 4, 2001
6 89
7 87
8 92
9 86
10 93
(arom. Cqu,t); 132.26 (arom. Cqu,t); 136.21 (arom. Cqa); 140.29 (arom. Cqu,);
146.49 (atom. Cqut); 170.93 (N-C=N). Analysis (C44H52N4S2Ge): Calcd. %: C,
68.16; H, 6.71; N,27.23. Found %" C, 68.22; H, 6.87; N, 7.19.
1R p-CH3-C6H4; R CH3; 3R H; M Ge
IH-NMR (CDCI3)" 0.99 (s, 3H, CH_.3Ge); 1.73 (d, 6H, J 6.8 Hz, CH_H_3-CH);
2.24-2.58 (m, 7H, p-CH_0_3 and CH,S); 3.06-3.73 (m, 12 H, CH_N); 4.5,1 (q, 2H,
J 6.8 Hz, CH-CH3); 6.85-8.--8 (m, 18 H, C6H_4 and CoI-I7). '3C-NMR
(CDCI3)" 9.06 (CH3-Ge); 19.97 (p-CH3); 21.61 (CH3-CH); 29.70 (C..C_H_S); 34.32
(CH-CH3); 45.84 (CH2N); 46.02 (CH2N); 47.26 (CH2N); 123.20 (aromatic
C_H); 125.20 (atom. CH); 125.78 (atom. CH); 128.62 (arom. C_H); 128.89
(atom. CH); 129.22 (atom. CH); 129.36 (atom. C__H); 129.50 (atom. C__H);
132.23 (atom. CH); 133.81 (atom. Cquat); 134.07 (atom. Cquat); 138.26 (atom.
Cq,t); 141.35 (atom. Cq,0; 147.32 (atom. Cqu,); 163.09 (N-C_=N). Analysis
(C42H4sN4S2Ge)' Calcd. %" C, 67.51; H, 6.43; N, 7.50. Found %: C, 67.44; H,
614 7.47.
--'8;2 '-" n-C6H3," R CH3; M Si
H-NMR (CDCI3)" 0.81 (t, 6H, J 5.9 Hz, CH...3CH2); 1.01-1.44 (rn, 26H,
(CH_). and CH_.,-CHS); 1.75 (d, 6H, J 6.9 Hz, CH_H_3-CH); 2.36-2.79 (rn, 2H,
CH3-CH.S); 3.04-3.81 (m, 12H, CH.0_:N); 4.53 (q, 2H, J 6.9 Hz, CH-CH3);
7.25-7.56 (m, 8H, C0H7); 7.61-7.86 (m, 4H, Col-tT); 7.90-8.21 (m, 2H,
CloH7). 3C-NMR (CDC): 14.12 (CH3CH2); 16.18 (CH2Si); 18.48 (CHsCH:);
18.67 (CH3CH); 19.58 (CHS); 20.85 (CH3CH); 22.56 (CH3CH_CH2); 25.58
(CH3CHzCHzCH:z); 31.58 (CHzCH:Si); 33.44 (.G,.H-CH3); 44.40 (,C__H_N); 46.16
(CH2N); 50.13 (CH2N); 123.18 (aromatic CH); 125.38 (arom. CH); 125.79
(arom. CH); 125.99 (arom. C__H); 127.02 (arom. C__H); 128.71 (arom. CH);
128.87 (arom. C_.H); 130.96 (arom. Cqat); 133.68 (atom. Cq,t); 134.08 (arom.
Cq,,t); 173.80 (N-C=N). Mass Spectrum: m/z 792 [M]+; 704 [M 2x44]+.
Analysis (C48H68N4S.,Si)' Calcd. %: C, 72.73; H, 8.59; N, 7.07. Found %' C,
7.69;RH, 8.65; N,
733.
n-C6HI3; H; M Si
H-NMR (CDCI3): 0.83 (t, 6H, J 5.7 Hz, CH.._CH2); 1.00-1.49 (m, 20H,
(CH.._2)5); 1.75 (d, 6H, J 6.3 Hz, CH__3-CH); 2.36-2.84 (m, 4H, CH__S); 2.95-
4.10 (m, 12H, CH_0_2N); 4.56 (q, 2H, J 6.3 Hz, CHCH3); 7.28-7.63,,(m, 8H,
C.ol-t7); 7.64-7.88 (m, 4H, C0_HT); 7.92-8.26 (m, 2H, CioH__7). '"C-NMR
(CDCI3)" 14.13 (.G_H3CH2); 16.21 (C_C_H2Si); 18.51 (CH3CH2); 19.32 (CH2S);
22.59 (C_.H3-CH); 29.70 (CH3CH:CH2); 31.61 (CH3CH2CH,C__H_); 32.66
(CH:CH2Si); 34.22 (CH-CH3); 46.02 (CH2N); 49.88 (C__H2N); 49.99 (C__H2N);
123.28 (aromatic C__H); 124.75 (atom. _G.H); 125.39 (atom. C_.H); 125.81 (atom.
C_H); 126.50 (atom. C__H); 127.03 (atom. C__H); 128.87 (atom. C_H); 130.05
(atom. Cqo,3; 131.03 (atom. Cquat); 133.77 (arom. CqL,,0; 176.76 (N-C__=N).
Analysis (C46H64N4S2Si): Calcd. %: C, 72.25; H, 8.38; N, 7.33. Found %" C,
72.29; H, 8.50; N, 7.14.
R
R -R i-C.HII, CH3 M Si
IH-NMR (CDCI3)" 0.86 (d, 12H, J 5.6 Hz, (C._)2CH); 1.04 1.47 (rn, 16H,
CH_.2CH__:CH and CH__3CHS); 1.71 (d, 6H, J 6.6 Hz, CH__3-CH); 2.31 2.97 (m,
2H, CH__S); 3.13 3.84 (m, 12H, CH_.2N); 4.60 (q, 2H, J 6.7 Hz, CH-CH3);
7.21 7.61 (m, 8H, CoI-t7); 7.69 7.96 (m, 4H, CoH7); 8.01 8.10 (m, 2H,
C,o7). 13C-NMR (CDCI3): 18.19 (CHzSi); 18.92 (CHS); 21.15 ((C__H3):CH);
21.26 (CH3-CH); 22.15 (C_.H3-CH); 30.85 ((CH3)zCH); 32.14 (CHzCH); 34.27
(CH-CH3); 50.69 (CHzN); 51.82 (CHzN); 53.44 (CHzN); 123.04 (aromatic
CH); 124.89 (atom. CH); 125.70 (atom. CH); 126.03 (atom. CH); 126.56
(atom. CH); 127.99 (atom. C_G_H); 128.39 (atom. C__H); 131.20 (atom. Cqat);
133.91 (atom. Cq,at); 135.83 (atom. Cqat); 172.32 (N-C=N). Analysis
(C46H64N4S_Si): Calcd. %: C, 72.25; H, 8.38; N, 7.33. Found %: C, 72.17' H,
8.45; N, 7.29.
R=-'R=i-C.HI; R=H; M=Si
H-NMR (CDCI3): 0.86 (d, 12H, 5.6 Hz, (CH...)_CH); 1.07 1.46 (rn, 10H,
CH_..,CH_.:CH); 1.68 (d, 6H, 6.9 Hz, CH...3-CH); 2.37 2.79 (rn, 4H, CH._...S);
3.13 3.81 (m, 12H, CH..N); 4.63 (q, 2H, J 6.7 Hz, C_H-CH3); 7.23 7.61
(m, 8H, C0I-tv); 7.66- 7.90 (m, 4H, C0I-tv); 8.00 8.11 (m, 2H, CtoH__7). 3C-
NMR (CDCI3): 17.36 (CHzSi); 18.94 (CHzS); 20.31 ((CH3),,CH); 22.17 (CH3-
CH); 30.86 ((CH3)zCH); 32.37 (CHzCH); 34.36 (C_.H-CH3); 50.03 (CHzN);
50.24 (CHzN); 51.11 (CH:N); 123.81 (aromatic CH); 124.95 (atom. CH);
201
Ghassoub Rima et al. Radioprotective Activity ad Synthesis ofSiladithioacetals and Germadithio
Acetals Derivedfrom N-Substituted Napthylethylemidazoline
11 87
12 90
125.56 (arom. CH); 126.34 (arom. CH); 126.81 (arom. CH); 127.74 (arom.
CH); 128.89 (atom. CH); 131.29 (arom. Cquat); 133.93 (atom. Cqu,t); 136.53
(arom. Cquat); 171.81 (N-C=N). Analysis (C44H60N4SzSi): Calcd. %: C, 71.74;
H, 8.15; N, 7.61. Found %" C,, 71.81; H, 8.19; N, 7.56.
R p-CH3-C6H4; 'R CH3; 'R CH3; M Si
H-NMR (CDCI3)" 0.97 (s, 3H, CH__3-Si); 1.38 (d, 6H, J 6.8 Hz, CH__3-CH-S);
1.71 (d, 6H, J 6.9 Hz, CH.__-CH); 2.23-2.56 (m, 5H, p-CH_.3 and CH-S); 3.12-
3.70 (m, 12 H, CH_.zN).,; 4.58 (q, 2H, J 6.9 Hz, CH-CH3); 6.84-8.19 (m 18
H, C6H_4 and C0HT). 'C-NMR (CDCI3): 9.07 (CH3-i); 18.84 (p-CH3)" 1.64
(CH3-CH); 20.19 (CH3-CH); 21.64 (CH-CH3); 33.99 (CH-CH3); 44.51 (CH2N);
44.98 (CHzN); 46.29 (.C,.H2N); 124.96 (aromatic CH); 125.84 (atom. CH);
126.02 (arom. CH); 127.27 (atom. CH); 127.69 (atom. CH); 128.42 (atom.
CH); 129.06 (arom. CH); 129.54 (atom. CH); 133.16 (arom. CH); 133.50
(arom. Cqua,); 134.12 (arom. Cquat); 136.31 (atom. Cqat); 140.13 (atom. Cqa,);
143.39 (atom. Cq,t); 171.85 (N-C=N). Analysis (C44H.zN4S2Si): Calcd. %: C,
72.53; H, 7.14; N, 27.69. Foun,d %: C, 72.47; H, 7.21; N, 7.76.
'R p-CH3-C6H4; R= CH3; 'R H; M Si
H-NMR (CDCI3)' 1.03 (s, 3H, CH_.3-Si); 1.72 (d, 6H, J 7.1 Hz, CH.O_3-CH);
2.24-2.60 (m, 7H, p-CH__3 and CH__2S); 3.08-3.66 (m, 12 H, CH___zN); 4.5,[ (q, 2H,
J 7.1 Hz, CH-CH3); 6.85-8.11 (m, 18 H, C6H_.4 and CIoH7). 'C-NMR
(CDCI3)" 8.88 (CH3-Si); 18.79 (p-CH3); 19.54 (CH3-CH); 22.61 (CHzS); 34.09
(CH-CH3); 44.96 (CHzN); 45.09 (CHzN); 46.13 (CHzN); 124.18 (aromatic
CH); 125.69 (arom. CH); 126.19 (atom. CH); 128.41 (arom. C_.H); 129.12
(atom. CH); 129.81 (atom. C_.H); 130.79 (arom. _C.H); 131.46 (atom. CH);
133.18 (arom. CH); 134.18 (arom. Cqa,); 134.41 (arom. Cqa,); 136.74 (arom.
Cqoat); 141.08 (arom. Cquat); 144.38 (arom. Cq,,t); 172.01 (N-C=N). Analysis
(C42H48N4SzSi)" Calcd. %" C, 72.00; H, 6.86; N, 8.00. Found %" C, 72.11; H,
6.83; N, 8.06.
Melting points were taken uncorrected on a Leitz Biomed hot-plate microscope apparatus or, in capillary
tubes, on a digital Electrothermal apparatus. Elemental analyses (C, H, N) were performed at the "Laboratoire
de Microanalyse de l'Ecole Nationale Sup6rieure de Chimie" of Toulouse.
Synthesis of metalladithioacetals 1-12
All these compounds were prepared by a method already described.
Siladithioacetal 8 (method A)
To a stirred mixture of freshly distilled triethylamine (0.96 g, 9.5 retool) and l-(l-thioethyl)-2-[1-
(1-naphthyl)ethyl]-2-imidazoline (2.70 g, 9.5 retool) in 30 ml of THF was added dropwise with stirring a
solution of diisoamyldichlorosilane (1.28 g, 4.75 mmol) in 30 ml of THF. The reaction mixture was
refluxed under an argon atmosphere for 5 hours. After filtration, the residue was concentrated under vacuum
to give 8 (3.2 g, 88 %).
Germadithioacetai 4 (method B)
A solution of bis(diethylamino)diisoamylgermane (1.71 g, 4.75 mmol) in 30 ml of THF was added
slowly with stirring to a solution of 1-(1-thioethyl)-2-[ l-(1-naphthyl)ethyl]-2-imidazoline (2.70 g, 9.5 mmol)
in 30 ml of THF. The reaction mixture was refluxed under argon atmosphere for 5 hours. After cooling to
room temperature, the volatile material was removed under vacuum to afford 4 (3.38 g, 91%).
The physiochemical data and the analyses are reported in Table
Synthesis of selenodiazadihexylgermetane 13
Method C
To a solution of 1,3-bis(trimethylsilyl)selenourea (2.2 g, 8.23 mmol) in 100 ml of THF was added
slowly with stirring a solution of bis(diethylamino)dihexylgermane (3.18 g, 8.23 mmol) in 10 ml of THF.
The reaction mixture was refluxed for 5 hours. After the mixture was cooled to room temperature the
volatiles were removed under vacuum to afford 13 (1.88 g, 45 %) as a yellow oil.
Method D
To a solution of 1,3-bis(trimethylsilyl)selenourea.(2.2 g, 8.23 mmol) and triethylamine (1.67 g,
16.46 mmol) freshly distilled in 100 ml of THF was added dropwise with stirring a solution of
dichlorodihexylgermane (2.58 g, 8.23 mmol) in 10 mi of THF. The reaction mixture was refluxed for 5
hours. After cooling to room temperature, the mixture was filtered and the volatiles were removed under
vacuum to give 13 (1.59 g, 38 %).
13" H-NMR (CDCI3): 0.15 (s, 18H, (CH__3)3Si); 0.86 (t, 6H, J 5.6 Hz, CH___CH2); 1.10-1.50 (m, 20H,
(CH_2)). '3C-NMR (CDCI3): 1.06 ((CH3)3Si); 14.08 (CH3CH2); 22.57 (CH3CH2); 25.27 (CH.CH2CH2);
25.76 (CH3CH2CH2CH2); 31.39 (CH2CH2Ge); 32 13 (CHzGe); C=Se not detected.
Mass spectrum' m/z 508 [M]/; 431 [M Se + i]/. Analysis (CgHaaN2SeSi2Ge)' Calcd. %" C, 44.79; H,
8.64; N, 5.50. Found %' C, 44.86; H, 8.69; N, 5.49.
202
Metal Based Drugs Vol. 8, No. 4, 2001
Synthesis of selenodiazadiisoamyigermetane 14
Using the same experimental procedure as for the synthesis of 13, the reaction of
dichlorodiisoamylgermane (3.13 g, 10.96 retool) in the presence of triethylamine (2.22 g, 21.92 retool)or
bis(diethylamino)diisoamylgermane (3.93 g, 10.96 retool) with 1,3-bis(trimethylsilyl)selenourea (2.93 g,
10.96 retool) does not lead to 14 but afford only the selenagermaadamantane 15 (2.50 g, 87 %) as a yellow
solid, and 1,3-bis(trimethylsilyl)carbodiimide.
Synthesis of hexaselenatetrakis(isoamylgerma)adamantane 15
A solution of LiEt3BH (57.20 mmol in 57.2 ml of THF) was added dropwise to elemental
selenium (2.26 g, 28.60 retool) via syringe. The mixture was stirred for hour at room temperature. A
solution of trichloroisoamylgermane (4.77 g, 19.07 retool) in 25 ml of anhydrous THF was added at 0 C
for hour. After the addition the reaction mixture was warmed to room temperature and stirred until the red
colour of the selenium salt had disappeared (5 days). The solvent was removed in vacuo and the residue was
extracted with toluene. After filtration and concentration the residue solid was crystallized from pentane to
afford 15 (4.5 g, 90 %).
15" H-NMR (CDCI3): 0.90 (d, 24H, J 5.4 Hz, (CH__)2CH); 1.20-2.11 (m, 20H, CH_.CH__CH). 1C-NMR
(CDCI): 21.96 (CH); 29.70 (C__H); 31.37 (C__H2); 32.69 (CH2). Mass spectrum (CI" C4): m/z 1079 [M +
29]t. Analysis (C20H4Se6Ge): Calcd. %" C, 22.90; H, 4.20. Found %: C, 22.93; H, 4.2..
This compound has been shown by X-Ray crystallographic methods to have an adamantane-type structure
but the quality of crystal does not permit us to do a structure refinement.
Synthesis of 2-[ l-(l-naphthyl)ethyll-2-imidazoline 16
a) N-Boc 2-(l-naphthylmethyl)-2-imidazoline
To a stirred solution of 2-(l-naphthylmethyl)-2-imidazoline (19 g, 90.35 mmol) in 200 ml of THF
was added dropwise a solution of ditertbutyldicarbonate (21 ml, 91.64 mmol) in 20 ml of THF. The
mixture was stirred at room temperature for 8 hours. The volatiles were removed under vacuum to afford N-
Boc 2-(1-naphthylmethyl)-2-imidazoline (25.52 g, 91%).
H-NMR (CDCI3)' 1.35 (s, 9H, (CH__3)3C); 3.76 (s, 4H, CH_.zN); 4.49 (s, 2H, CH_.-C,0HT); 7.33-7.92 (m, 7H,
C 10H7).
b) N-Boc 2-[lo(l-naphthyl)ethyl]-2-imidazoline
To a stirred solution of N-Boc 2-(1-naphthylmethyl)-2-imidazoline (25.52 g, 82.22 mmol) in 300
ml of THF, at-78 C, was added dropwise a solution of n-butyllithium 1.6 M in hexane (51.4 ml, 82.22
mmol). The mixture was stirred at this temperature for 3 hours. A solution of methyliodide (5.9 ml, 94.9
retool) in 30 ml of THF was added dropwise. The mixture was then stirred at room temperature for a night.
The volatiles were removed in vacuo and the residue was extracted with of a mixture ether/pentane (1/1, 400
ml). After filtration, the solvent was removed under reduced pressure to afford N-Boc 2-[1-(1-naphthyl)ethyl]-
2-imidazoline (23.86 g, 90 %).
'H-NMR (CDCI3): 0.99 (s, 9H, (CH__3)3C); 1.41 (d, 3H, J 7.0 Hz, CH_.3-CH); 3.57 (s, 4H, CH__2N); 5.15 (q,
1H, J 7.0 Hz, CH-CH3); 7.20-7.90 (m, 7H, C,0HT). '3C-NMR (CDCI3)" 19.09 (CH3-CH); 27.87 ((CH3)3-
C); 35.80 (CH-CH3); 47.79 (CHz-N); 51.40 C.C,.Hz-N); 83.05 ((CH3)3-C); 123.01 (aromatic CH); 123.30
(atom. CH); 125.64 (atom. C_.H); 125.75 (atom. CH); 126.36 (atom. CH); 127.62 (atom. CH); 128.80
(atom. CH); 131.08 (atom. Cquat); 133.89 (atom. Cqu,t); 138.10 (arom. Cqo,t); 150.72 (atom. Cqat); 165.42
(N-C=N). Mass spectrum: m/z 324 [M]
c) 2-[1-(1-naphthyl)ethyl]-2-imidazoline
To a stirred solution ofN-Boc 2-[1-(1-naphthyl)ethyl]-2-imidazoline (23.86 g, 74.00 mmol) in 150
ml of acetic acid was added dropwise a solution of chlorhydric acid (10 ml, 36%). The mixture was stirred at
40 C for 5 hours. The mixture was diluted with 400 ml of water and the aqueous layer was extracted with
dichloromethane (4x150 ml). The organic layer was washed with a solution of NaOH (25 %). The organic
layer was then dried over NazSO4. Evaporation of solvent in vacuo gave 16 (8.63 g, 52 %). mp: 160-161 C.
16: H-NMR (CDCI): 1.70 (d, 3H, J 7.1 Hz, CH._-CH); 3.53 (s, 4H, CH_..N); 4.39 (q, IH, 7.1 Hz,
CH-CH3); 7.09 (s, H, Nil); 7.40-7.61 (m, 4t-I, C0_.H7); 7.60-7.81 (m, 2H, C,0_.H7); 7.82-8.15 (m, 1H,
CI-t7). '"C-NMR (CDCI3): 18.95 (CH3-CH); 35.50 (CH-CH3); 47.76 (CH2-N); 123.08 (atom. CH); 124.61
(atom. CH); 125.63 (atom. C_.H); 125.96 (atom. CH); 126.67 (atom. CH); 128.13 (atom. CH); 129.00
(atom. CH 131.24 (atom. Cqat); 133.87 (atom. Cqat); 136.71 (arom. Cquat); 171-80(N-C=N). I.R. (cm):
VNH 330 Mass spectrum m/z223 [M o 80.3--
-1] Analysis (CIsHI6N2): Calcd. Vo: C, 6; H, 7.14 N, 12.50.
Found %" C, 80.70; H, 7.21; N, 12.09.
Synthesis of l-(l-thioethyl)-2-[ I-(1-naphthyl)ethyl]-2-imidazoline 17
A solution of 2-[1-(1-naphthyl)ethyl]-2-imidazoline 16 (3.66 g, 16.32 retool) in 60 ml of dry
toluene was mixed with a solution of ethylene sulfide (1.15 ml, 19.58 retool) in 20 ml of dry toluene
(sealed tube, argon flushed). The reaction mixture was then heated (110 C oven) for 22 hours. After cooling,
100 ml of cold diethyl ether was added with stirring to the reaction mixture and a small amount of
polyethylene sulfide was filtered. The solvent was removed under reduced pressure to give a yellow pasty
product 17 (3.25 g, 70 %).
17: H-NMR (CDCI.): 1.68 (d, 3H, J 6.8 Hz, CH..._-CH); 2.43 (s, 1H, SH); 2.54-2.84 (m, 2H, CH__2-S);
3.02-3.91 (m, 6H, CH__2N); 4.41 (q, 1H, J 6.8 Hz, CH-CH3); 7.38-7.50 (m, 4H, C0__H7); 7.58-7.79 (m, 2H,
203
Ghassoub Rima et al. Radioprotective Activity ad Synthesis ofSiladithioacetals and Germadithio
Acetals Derivedfrom N-Substituted Napthylethylemidazoline
C,oH7); 7.80-8.13 (m, 1H, C,oH7). 3C-NMR (CDC]3): 19.09 (CH3-CH); 23.40 (CH2-S); 35.12 (CH-CH3);
48.85 (H2-N); 49.09 (CH2-N); 52.41 (.G_H2-N); 122.81 (arom. CH); 124.54 (arom. CH); 125.43 (arom.
C__H); 125.80 (arom. CH); 126.09 (arom. _C_H); 127.78 (arom. CH); 129.!2 (arom. C_.H); 131.04 (arom. Cqua,);
133.85 (argm. Cquat); 136.67 (arom. Cqua,); 168.36 (N-.Q=N). I.R. (cm"): vsH 2542. Mass spectrum" m/z
283 [M-I]'-. Analysis (CI7H2oN2S): Calcd. %: C, 71.83; H, 7.04; N, 9.86. Found %: C, 71.89; H, 7.17; N,
9.77.
Synthesis of l-(2-thiopropyl)-2-ll-(1-naphthyi)ethyl]imidazoline 18
Using the same experimental procedure as for the synthesis of 17, the reaction of 16 (3.66 g, 16.32
mmgl with propylene sulfide (1.29 ml, 16.40 mmol) leads to a yellow pasty product 18 (3.17 g, 65 %).
18: 'H-NMR (CDCI3): 1.24 (d, 3H, J 6.6 Hz, CH_-CH); 1.65 (d, 3H, J 6.9 Hz, CH..._-CH); 2.48 (s, 1H,
SH); 2.58-2.86 (m, 1H, C_.H-CH3); 3.06-4.02 (m, 6H, CH__zN); 4.46 (q, 1H, J 6.9 Hz, CH-CH3); 7.11-7.52
(m, 4H, CoH7); 7.66-7.82 (m, 2H, CIoHT); 7.84-8.16 (m, 1H, C10H7). 3C-NMR (CDCi3): 19.13 (C__.H3-CH);
21.75 (CH-CH3); 23.08 (CH3-EH); 35.17 (CH-EH3); 48.89 (CH2-N); 49.21 (CH2-N); 52.46 (CH2-N); 123.01
(arom. CH); 124.61 (arom. CH); 125.51 (arom. CH); 125.90 (arom. CH); 126.13 (arom. CH); 127.83
(arom. CH); 129.19 (arom. CH); 131.09 (arom. Cquat); 134.06 (arom. Cquat); 137.02 (arom. Cquat); 168.61
(N-C=N). I.R. (cm): VsH 2548. Mass spectrum: m/z 297 [M-1]T. Analysis (CsH_zN_S)" Calcd. %: C,
72.48; H, 7.38; N, 9.40. Found %: C, 72.57; H, 7.61; N, 9.32.
Synthesis of 1, 3-bis(trimethylsilyi)selenourea 19
To a stirred solution of selenourea (10 g, 81.29 mmol) and triethylamine (36 ml, 186.96 mmol) in
200 ml of THF was added dropwise a solution of chlorotrimethylsilane (20.6 ml, 81.29 mmol) in 50 ml of
THF. The reaction mixture was refluxed under an argon atmosphere for 8 hours. After filtration, the residue
was concentrated in vacuo to afford 19 (18.47 g, 85 %). mp: 146-148 C (dec.).
13
19: H-NMR (CDCI3): 0.35 (s, 18H, (C..l_)3Si);+ 6.20 (s, 2H, Nil_). C-NMR (CDCI3): 1,.02, ((CH3)3Si);
C=Se not detected. Mass spectrum: m/z 267 [M] Analysis (C7Hz0N2SeSi2): Calcd. %: C, .1.4; H, 7.46;
N, 10.45. Found %: C, 31.41; H, 7.51; N, 10.41.
Pharmacology: evaluation of radioprotection
Three- month-old male mice (Swiss, France), 25 g body weight, were used.
The radioprotective effect of compounds was evaluated by determining the dose reduction factor
(DRF), defined as the ratio of 50% lethal-dose irradiation 30 days (LDs0/30 days) of injected mice to that of
control mice. Initially the survival rate was determined 30 days after irradiation in different groups of 10
mice receiving an intraperitoneal (i.p.) injection of the test compound with a dose equal to half or one-eighth
of its LDso 15 or 90 rain before whole-body irradiation delivered with a dose equal to the LDoo/30 days of
control mice (7.5, 7.75 or 8 Gy according to the irradiation date), or with a dose equal to this dose + 2 Gy.
When necessary, other irradiation doses were tested in order to evaluate the irradiation LD_o of protected mice
by the Kraber method (calculated or graphic). [19]
The radiosensitivity of the strain was regularly monitored by the determination of lethality curves of
males and females. The LD0/30 days was between 6.5 + 0.3 and 6.75 + 0.3 Gy according to the date (P <
0.05). Under these conditions significant protection was observed with a DRF value superior to 1.15.
The toxicity was evaluated by a Probit analysis of the LD0, [20-21] the dose range being
determined in a preliminary study. Five groups of ten mice were then injected with different doses within
this range.
RESULTS AND DISCUSSION
Synthesis of metalladithioacetais tR2RMISCH(3R)CH2-NEI]2
NEI 2-[1-(1-naphthyl)ethyl]-2-imidazoline
The synthesis of sila- and germadithioacetals compounds derived from N-substituted 2-[1-(1-
naphthyl)ethyl]-2-imidazoline was realised by two methods, A and B [4, 22].
Method A
The action of the dichlorodiorganometallane [22] on two equivalents of N-substituted
naphthylimidazoline in refluxing anhydrous tetrahydrofuran in the presence of freshly distilled triethylamine
gave the acyclic derivatives (Scheme 1) in yields of 85-90 %.
R2RMCI2 2 HSCHCH2N, N w R RM CHCH2
1-12
Scheme
+ 2 EtsN,HC
204
Metal Based Drugs Vol. 8, No. 4, 2001
Method B
The reaction of two equivalents of N-substituted naphthylimidazoline with the
bis(diethylamino)dialkylmetallane in anhydrous tetrahydrofuran (a cleavage of M-N bonds by the SH groups)
[4, 22, 23] gave the corresponding organometallated derivatives (Scheme 2) in_,vield of 92-96 %.
IR2RM2)2 + 2 HS-'HCH2N .N IR2RM 82N .N
1-12
Scheme 2
M =Ge
R 2R n-C6Hi3; 3R CH3 (1)
R 2R n-C6Hi3; 3R H (2)
R 2R i-CsH; 3R CH3 (3)
R 2R i-CsHI; 3R H (4)
R p-CH3-C6H4, 2R CH3; 3R CH3(5)
R p-CH3-C6H4, 2R CH3; 3R H (6)
M=Si
R 2R n-C6Hl3; 3R CH3 (7)
R 2R n-C6Hl3; 3R H (8)
R 2R i-CsHil; 3R CH3 (9)
IR 2R i-CsHII; 3R H (10)
IR= p-CH3-C6H4, 2R CH3; 3R CH3(II)
IR= p-CH3-C6H4, 2R CH3; 3R H (12)
Synthesis of selenodiamgermetane R2Ge-N(SiMe3)(C=Se)N(SiMe3)
Case R n-C6H13
This compound was also synthesised by two methods, C and D.
Method C
Treatment of 1,3-bis(trimethylsilyl)selenourea, in stoichiometric amounts, with bis(diethyl-
amino)dihexylgermane in refluxing anhydrous tetrahydrofuran resulted with a cleavage of Ge-N bonds by the
NH groups (a transamination reaction) [22-26] forming the corresponding diazagermetane (Scheme 3) in yield
of 45 %.
(n-C6HI3)2Ge(NEt2)2 +
Me3Si(H)N--N(H)SiMe
Se
Me3i
THF, refluxed /N\
(n-C6H13)Je
\''" /C=Se
"2 Et2NH N
Me3Si
Scheme 3
Method D
The action of dichlorodihexylgermane, in stoichiometric amounts, on 1,3-bis(trimethyl-
silyl)selenourea, in refluxing anhydrous tetrahydrofuran in the presence of freshly distilled triethylamine gave
by a cyclization reaction, with elimination of hydrochloric acid from Ge-C1 and NH [22,26] group, the
corresponding product (Scheme 4) in yield of 38 %.
(n-C6H13)2GEC12 + Me3Si(H)N-
i-N(H)S iMe3
Se
Case R i-CsHl
Scheme 4
Me3
THF, Et3N /
/C Se
2 Et3N,HCI N
13
Me3Si
205
Ghassoub Rima et al. Radioprotective Activity ad Synthesis ofSiladithioacetals and Germadithio
Acetals Deri edfrom N-Substituted Napthylethylemidazoline
Using the same procedures described above, the reaction between bis(diethyl-
amino)diisoamylgermane or dichlorodiisoamylgermane and 1,3-bis(trimethylsilyl)selenourea does not lead to
stable heterocycle but gives only the selenagermaadamantane 15 and 1,3-bis(trimethylsilyl)carbodiimide
probably via the intermediate selenodiazagermetane. (Scheme 5).
(i-CsHI)2Ge(Y)2 +
Me3Si(H)N--N(H)SiMe3
Se
(Y= CI, NEt2)
THF, refluxed /N
(i'CsHI )2Ge. -_Se
-2YH
\
N
/
14
Me3Si
(i.CsH l)4Ge4Se6 Me3Si.N=C=N.SiMe3
15
Scheme 5
iMe
-..c c
(i-CsH)
--Se3.. (i-CsH N
1 SNe3
P
'.C,H,,Ge=
N--Se +
) Se
(i-c
_Nc
(i.c,,,) =N--Sc
14'
14
EMeSiN=C=Se
+ Mt3SiN=C=N--SiM
15
Scheme 6
Reaction mechanism
The formation of the selenagermaadamantane may be explained by two ways as follows (Scheme 6).
206
Metal Based Drugs Vol. 8, No. 4, 2001
Path A" The nucleophilic intramolecular attack of selenium on the germanium atom leads to a
corresponding intermediate 14'. Then, this cyclic intermediate 14' undergoes a decomposition to give
the germaneselone and 1,3-bis(trimethylsilyl)carbodiimide.
Path B: The ring cleavage of 14 results in the formation of the monomeric species [(i-
CsH)2Ge=N-SiMe3] and [Me3Si-N=C=Se] as an intermediate, which submits to a pseudo Wittig
reaction to afford the intermediate 14'.
The germaneselone derivative showed a rearrangement to give preferentially the
selenagermaadamantane 15 which has been completely characterised by usual spectroscopies, mass
spectrometry and X-ray diffraction structure.
Molecular modelisation study (ESSF field)
This study showed the very important steric bulk of the isoamyl groups. The heterocycle 13 seems
to benefit a protection of the reactive centre induced by the hexyl groups.
R i-CsH R n-C6Ha
Synthesis of selenagermaadamantane 15
The treatment of isoamyltrichlorogermane with lithium selenide gave tetragermahexaselenide 15
[27]. (Scheme 7). The structure was determined by X-ray crystallography.
4 (i-CH)GeCI + 6 LiSe THF
refluxed
(i-CsHil)
Se?ese
.Ge Ge
(/-CsH) / I'Se// \(i.CsHI)
Se/.-/Ge Se/
(i-C5H)
15
Sc heine 7
Synthesis of the 2-[l-(l-naphthyl)ethyl]-2-imidazoline 16
This product was synthesised according to the following reaction scheme. (Scheme 8).
207
Ghassoub Rima et al. Radioprotective Activity ad Synthesis ofSiladithioacetals and Germadithio
Acetals Derivedfrom N-Substituted Napthylethylemidazoline
Synthesis of 17 and 18
(Boc)O
THF, TA
Boc-OH
The stoichiometric addition of thiirane or methylthiirane to 16 in toluene at 110 C leads
respectively to l-(1-thioethyl)-2-[1-(1-naphthyl)ethyl]-2-imidazoline 17 and 1-(2-thiopropyl)-2-[1-(l-
naphthyl)ethyl]-2-imidazoline 18 (i.e. by a cleavage of the C-S bond by the NH group [28]) (Scheme 9) in
yields of 70 and 65%.
R=H
R Me 18
Scheme 9
Synthesis of 1,3-bis(trimethylsilyi)selenourea 19
The reaction between equimolar amounts of selenourea and chlorotrimethylsilane in the presence of
triethylamine in anhydrous refluxing tetrahydrofuran gives 19 (Scheme 10) in yield of 85 %.
H2N--C-NH + 2 Me3SiCI
Se
THF 2 Ft3N
Me3Si(H)N-ICI-N(H)SiMe + 2Et3N,HCI
Se 19
Scheme 10
208
Metal Based Drugs Vol. 8, No. 4, 2001
Table 2 Toxicity in mlce o'fsome ,,orgnlcndorgano,.,,m.eiiii'c derlv,aiiwe's
Compound LDs0 (mg/kg)
(mmol/k)
106
0.473
Me3Si(H)N-
-N(H)SiMo3
M31Si
(n-C6H)C(N\
N
Me3Si
50
0.238
80
0.106
212
0.271
185
0.221
150
0.192
600
1.150
Evaluation of the toxicity
The toxicity obtained with the different compounds has been summarized in table 2. The biological protocol
has been detailed in the Experimental section.
The 2-[1-(l-naphthyl)ethyl]-2-imidazoline (NEI) compounds seems to be less toxic than their 2-(l-
naphthyimethyl)-2-imidazoline (NMI) homologous. For example, NEI (16) show a two times lower molar
toxicity than NMI. The lower toxicity of organometallic compounds compared with basic materials is
confirmed by comparison between the toxicity of 13 and 19. Indeed compound 13 is eight times less toxic
(LD.o > 600 mg/Kg) than the starting derivative 19 (LDs0 > 75 mg/Kg). This means that the
organogermanium compound 13 have a toxicity four times lower than 19 one (expressed in mol fractions).
The results of the radioprotective activity will be reported at a later date.
209
Ghassoub Rima et al. Radioprotective Activity ad Synthesis ofSiladithioacetals and Germadithio
Acetals Derivedfrom N-Substituted Napthylethylemidazoline
Acknowledgements
The authors wish to thanks the D16gation G6n6rale pour l'Armement (D.G.A.), D6partement de
Chimie-Pharmacologie, Minist6re de la D6fense Nationale, France and we would also like to thank the
Comit6 de Radioprotection de I'EDF, France, for their financial support and interest in this research.
References.
1. H. Scheinin, R. Virtanen, E. Macdonald, R. Lammintausta, M. Scheinin, Prog. Neuro-
Psychopharmacol. Biol. Psychiatry, 13 (1989) 635.
2. M. B. Weinger, I. S. Segal, M. Maze, Anesthesiology, 71 (1989) 242.
3. Y. Amemiya, S. S. Hong, B. V. Venkataraman, P. N. Patil, G. Shams, K. Romstedt, D. R. Feller, F-L.
Hsu and D. D. Miller, J. Med. Chem., 35 (1992) 750.
4. M. Fatome, H. Sentenac-Roumanou, C. Lion, J. Satg6, M. Fourtinon and G. Rima, Eur. J. Med. Chem.,
19 (1984) 119.
5. M. Fatome, H. Sentenac-Roumanou, C. Lion, J. Satg6 and G. Rima, Eur. J. Med. Chem., 23 (1988)
257.
6. J. Satg, G. Rima, M. Fatome, H. Sentenac-Roumanou and C. Lion, Eur. J. Med. Chem., 24 (1989) 48.
7. G. Rima, J. Satg6, C. Lion, H. Sentenac-Roumanou and D. Guyot, Synth. React. lnorg. Met.-Org.
Chem., 19 (1989) 787.
8. G. Rima, J. Satg6, M. Fatome, J. D. Laval, H. Sentenac-Roumanou, C. Lion and M. Lazraq, Eur. J.
Med. Chem., 26 (1991) 291.
9. G. Rima, J. Satg6, H. Sentenac-Roumanou, M. Fatome, C. Lion and J. D. Laval, Eur. J. Med. Chem.,
28 ( 993) 761.
10. G. Rima, J. Satg6, H. Sentenac-Roumanou, M. Fatome, J. D. Laval, C. Lion, O. Alazard and
P.Chabertier, Appl. Organomet. Chem., 8 (1994) 481.
l. G. Rima, J. Satg6, H. Sentenac-Roumanou, M. Fatome, J. D. Laval, C. Lion and R. Dagiral, Appl.
Organomet. Chem., 0 (1996) 113.
12. G. Rima, J. Satg6, H. Sentenac-Roumanou, M. Fatome, J. D. Laval, C. Lion, C. Thiriot, R. Dagiral
and C. Martin, Main Group Met. Chem., 20 (1997) 255.
13. G. Rima, J. Satg6, R. Dagiral, C. Lion, Marc Fat6me, V. Roman and Jean-Denis Laval, Metal-Based
Drugs, 5 (1998) 139.
14. G. Rima, J. Satg6, R. Dagiral, C. Lion, H. Sentenac-Roumanou, M. Fat6me, V. Roman and J-D.
Laval, Metal-Based Drugs, 6 (1999) 49.
15. G. Rima, J. Satg6, R. Dagiral, C. Lion, Marc Fat6me, V. Roman and Jean-Denis Laval, Appl.
Organomet. Chem., 13 (1999) 583.
16. J. Satg6, Propri6t6s et applications biologiques de d6riv6s organom6talliques du silicium, du
germanium, et de l'6tain, rapport de mise au point A. E. P. A., juin 1981.
17. M.L. Patchen, T.J. Mac Wittie and J.F. Weiss, Int. J. Radiat.Oncol. Biol. Phys., 18 (1950) 286.
18. M.A. Saint-Martino-Descours, M. Cottin and H. Pacheco, Eur. J. Med. Chem. Chim. Ther., 14 (1979)
455.
19. J. Oiry, J. Y. Pue, J. L. Imbach, M. Fat6me, H. Sentenac-Roumanou and C. Lion, J. Med. Chem. 29
(1986) 2217.
20. P. Bonet-Maury and F. Patti, J. Radiol. Electrol. Arch. Electr. Med., 31 (1979) 455.
21. D. J. Finney, Probit analysis, Cambridge University Press, Cambridge (1947).
22. M. Lesbre, P. Mazerolles and J. Satg6 in: The Organic Compounds of Germanium, John Wiley and
Sons, New York, (1973).
23. J. Satg6 and M. Baudet, C. R. Acad. Sci. Paris. Set. C. 263 (1966) 435.
24. J. Satg6, A. Cazes, M. Bouchaut, M. Fatome, H. Sentenac-Roumanou and C. Lion, Eta'. J. Meal.Chem.,
17 (1982) 433.
25. G. Dousse, J. Satg6 and M. Rivi6re-Baudet, Synth. React. Inorg. Met.-Org. Chem., 3 (1973) I.
26. J. Satg6, M. Lesbre and M. Baudet, C. R. Acad. Sci. Paris. Set. C. 259 (1964) 4733.
27. M. Unno, Y. Kawai, H. Shioyama and H. Matsumoto, Organometallics, 16 (1997) 4428.
28. J. Corbin, K. F. Miller, N. Pariyadath, S. Wherland, A. E. Bruce and E. I. Stiefel, Inorg. Chim. Acta,
90 (1984) 41.
Received: December 13, 2001 Accepted: December 17, 2001
Accepted in publishable format: December 18, 2001
210

				

